# Ecotypic differentiation of leaf silicon concentration in the grass *Brachypodium hybridum* along a rainfall gradient

**DOI:** 10.3389/fpls.2024.1417721

**Published:** 2024-10-25

**Authors:** Susanne Kurze, Jinyu Ouyang, Florian Gade, Ofir Katz, Jörg Schaller, Johannes Metz

**Affiliations:** ^1^ Leibniz Centre for Agricultural Landscape Research (ZALF), Müncheberg, Germany; ^2^ Plant Ecology and Nature Conservation, Institute of Biology and Chemistry, University of Hildesheim, Hildesheim, Germany; ^3^ Dead Sea and Arava Science Center, Mount Masada, Tamar Regional Council, Israel; ^4^ Ben-Gurion University of the Negev, Eilat, Israel

**Keywords:** silicon concentrations, rainfall gradient, intraspecific variation, Mediterranean rangelands, local adaptation, slope exposure, ecotypes, *Brachypodium hybridum*

## Abstract

Ecotypic differentiation, reflected in substantial trait differences across populations, has been observed in various plant species distributed across aridity gradients. Nevertheless, ecotypic differentiation in leaf silicon concentration, known to alleviate drought stress in plants, remained hardly explored. Here, we provide a systematic test for ecotypic differentiation in leaf silicon concentration along two aridity gradients in the grass *Brachypodium hybridum* in Israel. Seed material was sampled in 15 sites along a macroclimatic aridity gradient (89 – 926 mm mean annual rainfall) and from corresponding north (moister) and south (more arid) exposed slopes (microclimatic gradient) at similar altitudes (mean north: 381 m a.s.l., mean south: 385 m a.s.l.). Plants were subsequently grown under common conditions and their leaf silicon concentration was analysed. Leaf silicon concentration increased with increasing aridity across the macroclimatic gradient, but did not differ between north and south slopes. The higher leaf silicon concentrations under more arid conditions can enhance the ability of plants to cope with more arid conditions by two mutually not exclusive mechanisms: (i) withstanding drought by reducing water loss and increasing water uptake or (ii) escaping drought by facilitating fast growth. Our study highlights that leaf silicon concentration contributes to ecotypic differentiation in annual grasses along macroclimatic aridity gradients.

## Introduction

Populations within a species can be adapted to specific, local environmental conditions, manifested in considerable intraspecific trait differences – a phenomenon termed ecotypic differentiation (Kawecki and Ebert, 2004). Ecotypic differentiation likely increases species’ vulnerability to environmental change, since each population is adapted to a narrower environmental range than the entire species (Davis and Shaw, 2001).

Ecotypic differentiation is common along natural aridity gradients, where water availability, growing season length, productivity, and competition intensity typically covary and increase in concert from the arid to the moister end of the gradient (Schiffers and Tielbörger, 2006; Metz and Tielbörger, 2016; Drori et al., 2021). Across macroclimatic aridity gradients, directional changes indicative of ecotypic differentiation have been observed in various morphological, physiological, and anatomical traits (e.g. Carlson et al., 2015; Kurze et al., 2017; Baughman et al., 2019; Metz et al., 2020). Differences in aridity at microclimatic scales, such as between corresponding north and south exposed slopes, can also lead to ecotypic differentiation, but findings are less consistent (Kurze et al., 2017; Nevo, 2021; Blanco-Sánchez et al., 2022; Wang et al., 2023).

Whether ecotypic differentiation along aridity gradients comprises leaf silicon concentration has remained nearly unexplored. Silicon is considered a quasi-essential nutrient in plants (Epstein, 1999) that has been shown to alleviate drought stress and may also influence the growth rate of plants and hence their competitive ability (Cooke and Leishman, 2011, Cooke and Leishman, 2016; de Tombeur et al., 2023). Assessing ecotypic differentiation in leaf silicon concentration is thus relevant for completing our understanding of ecotypic differentiation along aridity gradients.

Intraspecific differences in leaf silicon concentration or phytolith formation along gradients of water availability have been addressed only anecdotally (Katz et al., 2013; Issaharou-Matchi et al., 2016; Gao et al., 2019; de Tombeur et al., 2020; Kang et al., 2021). Leaf silicon concentration increased with lower water availability at macro- and microclimatic scales (Katz et al., 2013; Issaharou-Matchi et al., 2016; Kang et al., 2021). This pattern is in line with the assumption that higher leaf silicon concentration alleviates drought stress (Cooke and Leishman, 2016). However, also the opposite pattern has been observed, i.e. higher leaf silicon concentrations in plants from moister conditions, perhaps because higher water availability increases the availability of silicon to plants (Katz et al., 2013). Since all these studies have been conducted in the field, the observed differences in leaf silicon concentrations may be confounded by covarying environmental factors and cannot be attributed with certainty to genetic differences across populations (Kawecki and Ebert, 2004). To rigorously assess ecotypic differentiation in leaf silicon concentration and its underlying mechanisms, common garden experiments with plants originating from gradients of water availability are necessary.

Here, we systematically tested for ecotypic differentiation in leaf silicon concentration by studying intraspecific differences in *Brachypodium hybridum* Catalán, Joch. Müll., Hasterok & Jenkins, a common annual grass in the Mediterranean Basin, along two aridity gradients at different spatial scales in Israel. The macroclimatic aridity gradient ranged from arid to mesic-Mediterranean conditions, spanning 89 – 926 mm mean annual rainfall across 250 km. The microclimatic gradient consisted of north and south slopes across the macroclimatic gradient. Along the macroclimatic gradient, ecotypic differentiation has been shown for *B. hybridum* and other annual species in various traits, such as flowering time, plant size, and biomass allocation (e.g. Liancourt and Tielbörger, 2009; Kurze et al., 2017; Metz et al., 2020). Our study system is thus promising for testing ecotypic differentiation in leaf silicon concentration. Leaf silicon concentration was measured in plants grown under common, favourable conditions in a greenhouse to minimise confounding influences. We addressed the hypothesis whether leaf silicon concentration changed with aridity along both gradients.

## Materials and methods

### Study species

*Brachypodium hybridum* Catalán, Joch. Müll., Hasterok & Jenkins is a common annual grass in the Mediterranean Basin that inhabits a wide climatic range from arid to Mediterranean conditions, covering regions from approx. 100 to 1000 mm mean annual rainfall (López-Alvarez et al., 2015; Bareither et al., 2017). It is medium-sized (15-45 cm height) and occurs in the herbaceous layer of shrublands, pastures, and abandoned fields (Feinbrun-Dothan et al., 1998). In Israel, *B. hybridum* is the most abundant species of the *Brachypodium distachyon* s. l. complex, consisting of three species with different ploidy: *B. hybridum* (2n = 30), *B. distachyon* (2n = 10), and *B. stacei* (2n = 20) (Catalán et al., 2012; Bareither et al., 2017). Its wide distribution, ability of self-fertilisation, and the often high leaf silicon concentration of grasses (Hodson et al., 2005) render *B. hybridum* a suitable species for studying ecotypic differentiation in leaf silicon concentration.

### Sampling sites

**Figure 1:**
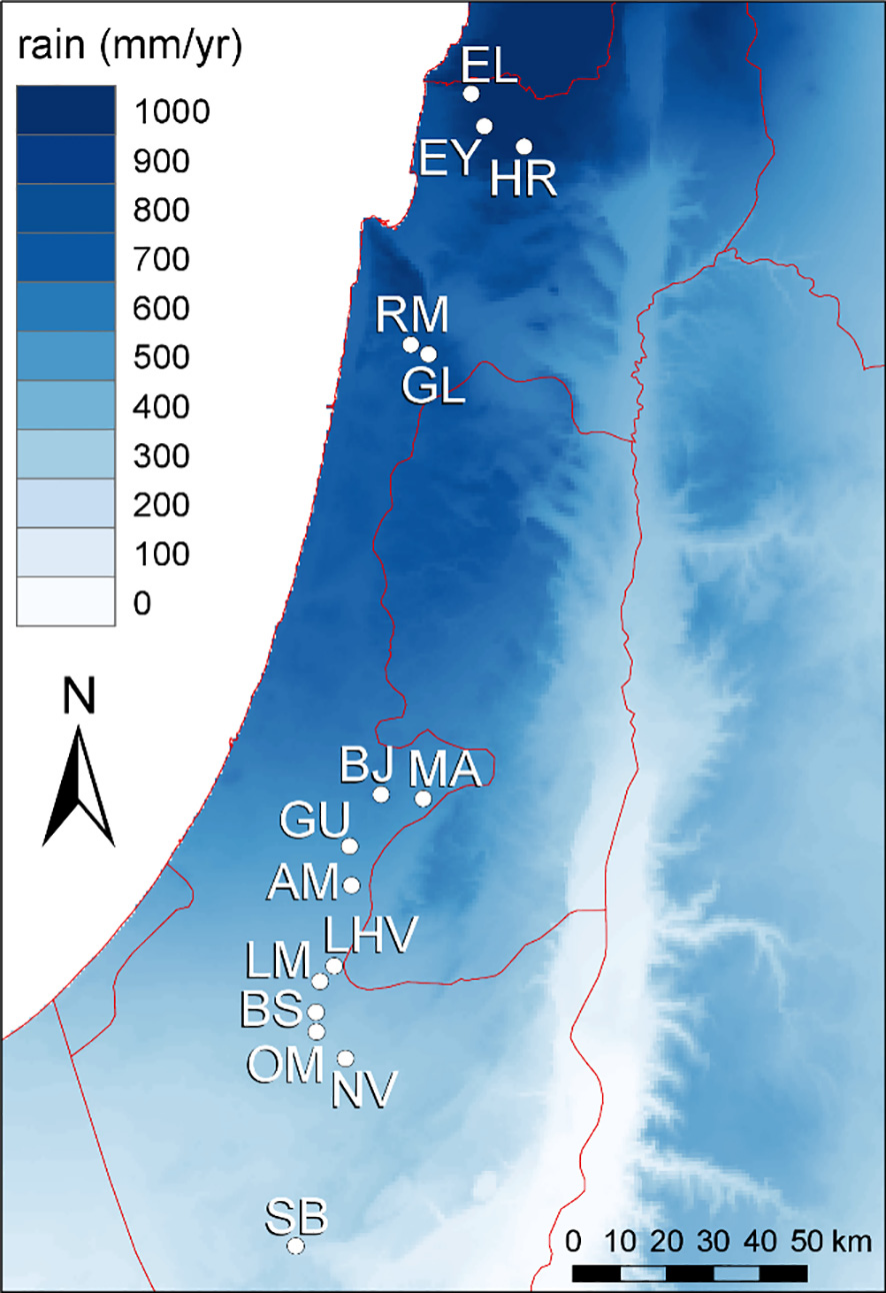
Mean annual rainfall in the study region and the location of the 15 sampling sites (for abbreviations see **Supplementary Table S1**) along the macroclimatic aridity gradient where mean annual rainfall decreases from North to South Israel.

Our macroclimatic aridity gradient comprised 15 sites (at least 4 km apart) across 250 km of a steep rainfall gradient from arid conditions in the south (mean annual rainfall 89 mm/year) to mesic-Mediterranean conditions in the north (926 mm/year) in Israel (**Figure 1**; **Supplementary Table S1**). The climate along the gradient is Mediterranean with hot, long, and almost rainless summers, and mild, wet winters. Growing season length increases from arid sites (3-4 months) to mesic-Mediterranean sites (6-7 months). Alongside, primary productivity and competition intensity decreases towards arid sites (Schiffers and Tielbörger, 2006; Metz and Tielbörger, 2016). All sites were situated on calcareous bedrock, experienced similar mean annual temperatures (17.8–19.8°C based on Worldclim), and harboured semi-natural shrublands or woodlands with herbaceous (mostly annual) plants dominating the inter-shrub matrix (see **Supplementary Table S1** for details). Rainfall data for each site was obtained from the nearest available rainfall station (usually < 3 km distance from sampling sites) of the Israel Meteorological Service (www.ims.gov.il) for the 35-year period prior to our research (1984–2020).

The microclimatic aridity gradient consisted of corresponding north and south exposed hill-slopes along the macroclimatic gradient. The opposite slopes were usually separated by a small valley (wadi) and lay 150-300 m apart. Bedrock, altitude, and inclination were similar for both slopes (see **Supplementary Table S1** for details). However, south slopes receive higher solar radiation in our study region resulting in higher evapotranspiration, lower soil moisture, and sparser vegetation than on north slopes (see **Supplementary Table S1**, Kutiel and Lavee, 1999; Nevo, 2021). These environmental differences render contrasting slopes suitable for testing ecotypic differentiation at small scales (Nevo, 2021).

### Seed material and plant cultivation

In spring 2019, seeds from 7-20 (mean 15, detailed numbers per site and slope in **Supplementary Table S1**) randomly selected mother plants (hereafter named genotypes) that were at least 3 m apart to diminish their genetic relatedness were collected within an area of 0.5 ha per site × slope combination. In the following winter, one plant per genotype was raised under common, favourable conditions in a greenhouse in Hildesheim, Germany, to diminish parental environmental effects.

For the present experiment, one offspring plant per genotype (i.e. 414 genotypes; 197 from north and 217 from south slopes; **Supplementary Table S1**) was raised under common conditions in the same greenhouse in Hildesheim from November 2020 to May 2021. Each plant was grown in a separate 400 ml pot (Deepot Cells, Stuewe & Sons, Oregon, US diameter 5.7 cm, depth 25 cm) filled with unfertilised silty clay from weathered loess near Hildesheim (pH 7.2 ± 0.1 mean ± standard deviation; 2.9 ± 0.1% soil organic carbon; 56 ± 16 ppm total nitrogen; 25.6 ± 4.9 mg plant-available silicon per kg soil dry weight based on CaCl_2_ extraction of 16 hours according to Schaller et al., 2021) that mimicked natural soil conditions in Israel (Zwikel et al., 2007). All pots received 40 ml water approx. once a week to provide favourable growth conditions. Temperature in the greenhouse ranged between 15-20°C (minimum 10°C) in winter and between 20-30°C (max. 35°C) in spring. Supplementary greenhouse LED lights adjusted day length to its natural course during the growth season in Israel. Plants from different sites and slopes were randomly arranged in the greenhouse and re-arranged every three weeks.

The experiment started on 29^th^ of November with the first irrigation and ended on 10^th^ to 14^th^ of May with harvesting all plants. At harvest, all individuals were mature with ripe seeds and showed signs of senescence. Plant biomass was separated into leaves, stems, reproductive organs, and roots. Leaves were dried for 24 h at 60°C.

### Silicon analysis

Silicon was extracted from grinded leaves (bulk sample) of each plant by an alkaline method using 30 mg of leaf material and 30 ml of 0.1 M sodium carbonate solution (Na_2_CO_3_). The solution was shaken in a water bath at 85°C for five hours and afterward filtered by a 0.2 μm syringe filter (ChromafilXtra CA-20/25). This method gives results comparable to complete digestion methods (Puppe et al., 2023). Silicon concentration of the leaf extract was determined with inductively coupled plasma optical-emission spectrometry (Varian, Vista-Pro radial, Palo Alto, California, USA).

### Statistical analysis

We tested whether leaf silicon concentration changed across both aridity gradients with a linear mixed model using the package lme4 (Bates et al., 2015) in R version 4.0.3 (R Core Team, 2020). Rainfall, slope, and their interaction were included as fixed explanatory factors, while site identity as random factor accounted for the missing independence of plants from the same sites. Significance of the fixed factors was assessed with type II Wald F-tests with Kenward-Roger approximated degrees of freedom in package car (Fox and Weisberg, 2019). The proportion of variance explained by the fixed factors (marginal R², R²_m_) and by both the fixed and random factors (conditional R², R²_c_) was calculated with package MuMIN (Barton, 2020).

## Results

**Figure 2:**
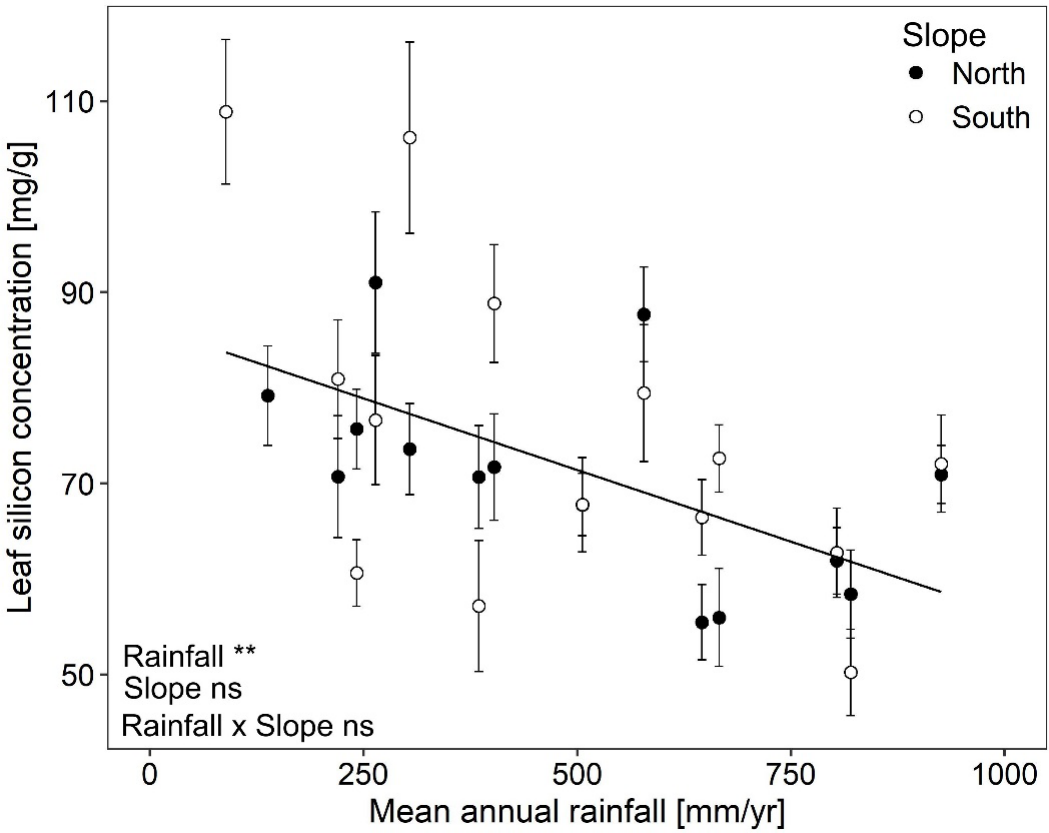
Leaf silicon concentrations (mean ± standard error) in *Brachypodium hybridum* along a macroclimatic (rainfall) and microclimatic (north vs. south slopes) aridity gradient. Significance levels are given with ** p < 0.01, ns not significant.

Leaf silicon concentration varied more than two-fold among *B. hybridum* plants originating from different site × slope combinations when grown under common conditions. Concentrations ranged from 50.2 mg/g ± 4.5 (mean ± standard error) to 108.9 mg/g ± 7.6 (**Figure 2**).

Leaf silicon concentration declined from drier to rainier sites along the macroclimatic aridity gradient (rainfall: F_1,13.4_ = 11.30, p = 0.005; **Figure 2**). This decline was similar for both slopes (rainfall × slope: F_1,399.1_ = 0.35, p = 0.554; **Figure 2**) and plants from north and south slopes exhibited overall comparable leaf silicon concentrations (slope: F_1,408.4_ = 2.40, p = 0.122; **Figure 2**). The whole linear mixed model, i.e. fixed and random factors together, explained 23% of the total variance (R²_c_ = 0.23) and the fixed factors alone accounted for 11% (R^2m^ = 0.11).

## Discussion

Our study points to ecotypic differentiation in leaf silicon concentration in *B. hybridum* along the macroclimatic aridity gradient. When grown under common conditions, plants originating from arid sites had higher silicon concentrations than conspecifics from more mesic sites. The magnitude of these ecotypic differences was comparable to interspecific differences among Poaceae species and larger than among dicotyledonous species with overall lower leaf silicon concentrations (Hodson et al., 2005).

Our finding of higher silicon concentration in plants from more arid conditions agrees with previous observations in both forbs and grasses (Katz et al., 2013; Issaharou-Matchi et al., 2016; Kang et al., 2021). Yet, these previous studies could not disentangle whether the observed patterns were due to genetically based ecotypic differences or reflected plastic responses to drier conditions. Our common garden experiment excluded plastic responses. It thus highlights that leaf silicon concentration can contribute to ecotypic differentiation along macroclimatic aridity gradients in annual grasses.

In annual plants, higher leaf silicon concentrations can enhance coping with more arid conditions by two mutually not exclusive strategies: withstanding or escaping drought. Higher silicon concentration can contribute to withstand drought, i.e. to maintain high performance under drought in the vegetative and reproductive phase, due to (i) enhanced water uptake via a higher resource allocation to roots, stronger osmotic driving force, or enhanced aquaporin activity (Chen et al., 2018), (ii) reduced transpirational water loss by a silicon double layer below the cuticle (Ma, 2004), and/or (iii) minimised oxidative damage (Cooke and Leishman, 2016). A pronounced escape strategy, i.e. growing faster and reproducing earlier to minimise the risk from late-season drought spells (Kooyers, 2015), is common in plants from arid conditions in various annual species (Kigel et al., 2011; Franks, 2011; Metz et al., 2020), and was also demonstrated for *B. hybridum* along our macroclimatic gradient (Kurze et al., 2017; Gade and Metz, 2024). High leaf silicon concentration may contribute to this escape strategy by facilitating growth rate because silicon can substitute carbon as structural component at cheaper metabolic costs (Raven, 1983; Cooke and Leishman, 2011; de Tombeur et al., 2023). The observed higher leaf silicon concentrations in plants from more arid conditions are thus likely adaptive.

However, we found no ecotypic differentiation in leaf silicon concentration between plants from north and south slopes, despite ample evidence for drier microclimates at south compared to north slopes (Kutiel and Lavee, 1999; Nevo, 2021). Similarly, little or no ecotypic differentiation between plants from north and south slopes emerged in other traits in *B. hybridum* along the same macroclimatic gradient (Kurze et al., 2017; Gade and Metz, 2024). Thus, plants from contrasting slopes likely constitute a single population within a site with sufficient geneflow among slopes to counteract microclimatic local adaptation (Blanco-Sánchez et al., 2022).

In conclusion, our findings showed that leaf silicon concentration can contribute to ecotypic differentiation in annual grasses along macroclimatic aridity gradients. This finding calls for further studies on intraspecific differences in leaf silicon concentration to explore the underlying mechanisms and using other species and environmental gradients to assess the prevalence and magnitude of this pattern. Considering the various functions of silicon in plants (Cooke and Leishman, 2016; de Tombeur et al., 2023), understanding intraspecific differences in silicon concentration may contribute to assess responses of plant species to environmental change.

## Data Availability

The raw data supporting the conclusions of this article will be made available by the authors, without undue reservation.
