# Supplementary material for: Ecotypic differentiation of leaf silicon concentration in the grass Brachypodium hybridum along a rainfall gradient
Source: Front Plant Sci. 2024 Oct 25;15:1417721. doi: 10.3389/fpls.2024.1417721 (PMC11544377; doi:10.3389/fpls.2024.1417721)
Supplement: Supplementary file 1 [file Table1.docx]

Supplementary Material

# Supplementary Tables

Table S1: Properties of the 15 sampling sites of *Brachypodium hybridum*, ordered from rainier to drier conditions along the macroclimatic aridity gradient in Israel. Site names refer to the nearest town. Mean annual rainfall (rain) and interannual variation of rainfall (CV rain; coefficient of variation in rainfall among individual years) for each site was obtained from the nearest station of the Israel Meteorological Service (www.ims.gov.il) calculated across the period 1984-2020. Mean annual temperature (temp.) per site was obtained from Worldclim (https://www.worldclim.org/). Rain, CV rain, and temperature describe the macroclimate and were not separately available for the microclimatic difference between north and south slopes. Altitude above sea level (a.s.l.) and inclination (incl., i.e. steepness of the slope) were similar between slopes, while vegetation was usually sparser at south slopes. The experiment included slightly variable numbers of genotypes (no. genotypes) per site × slope combination, as *B. hybridum* were scarce or even absent (NA) in some combinations.

| **site code** | **site** | **rain (mm/ yr)** | **CV rain (%)** | **temp. (°C)** | **slope** | **no. geno-types** | **altitude (m a.s.l.)** | **incl. (°)** | **GPS location** | **vegetation** |
| --- | --- | --- | --- | --- | --- | --- | --- | --- | --- | --- |
| HR | Harashim | 926 | 26 | 17.8 | N | 13 | 669 | 22 | 32.9646N, 35.3287E | dense woodland (c. 80 % tree cover) of *Quercus calliprinos*, *Calicotome villosa*, *Rhamnus lycioides*, *Pistacia lentiscus* |
|  |  |  |  |  | S | 15 | 750 | 20 | 32.9533N, 35.3269E | open woodland (c. 30 % tree cover, 30 % shrub cover) of *Quercus calliprinos*, *Calicotome villosa*, *Pistacia lentiscus* |
| EY | Ein Yaacov | 820 | 26.6 | 18.9 | N | 12 | 480 | 13 | 33.0057N, 35.2394E | dense woodland (c. 80 % tree cover) of *Quercus calliprinos*, *Calicotome villosa* |
|  |  |  |  |  | S | 17 | 490 | 12 | 33.0067N, 35.2394E | dense shrubland (c. 75 % shrub cover) of *Calicotome villosa*, *Sarcopoterium spinosum*, *Cistus* spp., *Quercus calliprinos* |
| EL | Eilon | 804 | 25.2 | 19.8 | N | 11 | 225 | 12 | 33.0688N, 35.2099E | woodland (c. 60 % tree cover) of *Quercus calliprinos*, *Calicotome villosa*, *Sarcopoterium spinosum*, *Salvia fruticosa* |
|  |  |  |  |  | S | 20 | 200 | 15 | 33.0697N, 35.2057E | open shrubland (c. 20 % shrub cover) of *Sarcopoterium spinosum* with single *Quercus* *calliprinos* trees (< 10 % cover) |

| **site code** | **site** | **rain (mm/ yr)** | **CV rain (%)** | **temp. (°C)** | **slope** | **no. geno-types** | **altitude (m a.s.l.)** | **incl. (°)** | **GPS location** | **vegetation** |
| --- | --- | --- | --- | --- | --- | --- | --- | --- | --- | --- |
| RM | Ramot Menashe | 666 | 27.7 | 19.7 | N | 19 | 158 | 15 | 32.5876N, 35.0613E | dense grassland (c. 70 % cover) of annual & perennial herbaceous vegetation, 10 % shrub cover of *Calicotome villosa* |
|  |  |  |  |  | S | 20 | 140 | 10 | 32.5885N, 35.0558E | similar to N-slope |
| GL | Gelad | 646 | 26.1 | 19.5 | N | 17 | 230 | 15 | 32.5677N, 35.1003E | dense grassland (c. 70 % cover) of annual & perennial herbaceous vegetation, 10 % shrub cover *Majorana syriaca*, *Calicotome villosa*, *Sarcopoterium spinosum* |
|  |  |  |  |  | S | 20 | 230 | 13 | 32.5703N, 35.0986E | similar to N-slope |
| MA | Mata | 578 | 29.3 | 18.1 | N | 17 | 606 | 17 | 31.7117N, 35.0691E | shrubland (c. 70 % shrub cover) of *Calicotome villosa*, *Sarcopoterium spinosum*, *Cistus* spp., *Quercus calliprinos*; herbaceous vegetation between shrubs |
|  |  |  |  |  | S | 20 | 610 | 17 | 31.7130N, 35.0665E | similar to N-slope, but only c. 60 % shrub cover |
| BJ | Bet Jimal | 506 | 30.7 | 19.8 | N | 16 | 335 | 19 | 31.7212N, 34.9735E | dense shrubland (c. 70 % shrub cover) of *Cistus* spp., *Quercus calliprinos*, *Salvia fruticosa*, *Pistacia* *lentiscus*, *Sarcopoterium spinosum* |
|  |  |  |  |  | S | 17 | 335 | 20 | 31.7232N, 34.9728E | shrubland (c. 50 % shrub cover) of *Sarcopoterium spinosum* |
| GU | Bet Guvrin | 403 | 31.4 | 19.8 | N | 11 | 302 | 14 | 31.6223N, 34.9009E | shrubland (c. 50 % shrub cover) of *Sarcopoterium spinosum*, *Pistacia lentiscus*, *Quercus calliprinos*, *Rhamnus lycioides* |
|  |  |  |  |  | S | 12 | 306 | 14 | 31.6238N, 34.9014E | similar to N-slope |
| AM | Amatziya | 385 | 27.5 | 19.6 | N | 12 | 329 | 19 | 31.5467N, 34.9041E | shrubland (c. 40 % shrub cover) of *Rhamnus lycioides*, *Pistacia lentiscus*, *Sarcopoterium spinosum*, *Quercus calliprinos* |
|  |  |  |  |  | S | 7 | 330 | 18 | 31.5480N, 34.9049E | open shrubland (c. 10 % shrub cover) of *Sarcopoterium spinosum* and single trees (*Quercus* *calliprinos*, *Rhamnus* *lycioides*); dense herbaceous vegetation between shrubs |

| **site code** | **site** | **rain (mm/ yr)** | **CV rain (%)** | **temp. (°C)** | **slope** | **no. geno-types** | **altitude (m a.s.l.)** | **incl. (°)** | **GPS location** | **vegetation** |
| --- | --- | --- | --- | --- | --- | --- | --- | --- | --- | --- |
| LHV | Lahav | 304 | 29.5 | 18.9 | N | 16 | 428 | 17 | 31.3914N, 34.8622E | shrubland (c. 70 % shrub cover) of *Sarcopoterium* *spinosum*, *Euphorbia hierosolymitana*; dense herbaceous vegetation between shrubs |
|  |  |  |  |  | S | 15 | 430 | 17 | 31.3925N, 34.8621E | shrubland (c. 40 % shrub cover) of *Sarcopoterium* *spinosum*, *Euphorbia hierosolymitana*; herbaceous vegetation between shrubs |
| LM | Lehavim | 264 | 32.7 | 19.1 | N | 15 | 340 | 20 | 31.3624°N, 34.8290°E | shrubland (c. 50 % shrub cover) of *Sarcopoterium* *spinosum*; herbaceous vegetation between shrubs |
|  |  |  |  |  | S | 15 | 340 | 20 | 31.3636N, 34.8288E | shrubland (c. 20 % shrub cover) of *Sarcopoterium* *spinosum*; loose herbaceous vegetation between shrubs |
| BS | Beer Sheva | 242 | 31 | 18.9 | N | 14 | 420 | 14 | 31.3037N, 34.8196E | open shrubland (c. 10 % shrub cover) of *Thymelea* *hirsuta*, *Sarcopoterium spinosum*; sparse herbaceous vegetation |
|  |  |  |  |  | S | 15 | 420 | 13 | 31.3066N, 34.8193E | open shrubland (c. 5 % shrub cover) of *Thymelea* *hirsuta*, *Sarcopoterium spinosum*; sparse herbaceous vegetation |
| OM | Omer | 220 | 29.3 | 19.2 | N | 13 | 342 | 17 | 31.2653N, 34.8192E | open shrubland (c. 10 % shrub cover) of *Thymelea* *hirsuta*, *Ballota undulata*, *Teucrium* spp.; mainly annuals between shrubs |
|  |  |  |  |  | S | 15 | 340 | 15 | 31.2641N, 34.8199E | open shrubland (c. 5 % shrub cover) of *Thymelea* *hirsuta*, *Ballota undulata*; mainly annuals between shrubs |
| NV | Nevatim | 138 | 37.5 | 19.2 | N | 15 | 381 | 15 | 31.2120N, 34.8840E | open shrubland (c. 25 % shrub cover) of *Echinops* spp.; mainly annuals between shrubs |
|  |  |  |  |  | S | NA | 380 | 15 | 31.2126N, 34.8840E | open shrubland (c. 10 % shrub cover) of *Echinops* spp.; mainly annuals between shrubs |
| SB | Sde Boqer | 89 | 42.3 | 18.8 | N | NA | 470 | 15 | 30.8528N, 34.7647E | open shrubland (c. 5 % shrub cover) with *Zygophyllum dumosum*, *Artemisia sieberi*, *Hammada scoparia*; sparse annual cover between shrubs |
|  |  |  |  |  | S | 14 | 470 | 15 | 30.8537N, 34.7639E | open shrubland (< 5 % shrub cover) with *Zygophyllum dumosum*, *Artemisia sieberi*, *Hammada* *scoparia*; sparse annual cover between shrubs |
